# Trace Minerals in Laying Hen Diets and Their Effects on Egg Quality

**DOI:** 10.1007/s12011-024-04121-8

**Published:** 2024-03-01

**Authors:** Shaaban S. Elnesr, Bothaina Y. Mahmoud, Paula Gabriela da Silva Pires, Priscila Moraes, Hamada A. M. Elwan, Nahed Ahmed El-Shall, Mohamed S. El-Kholy, Mahmoud Alagawany

**Affiliations:** 1 Department of Poultry Production, Faculty of Agriculture, Fayoum University, Fayoum, 63514 Egypt; 2 Department of Animal Science, Faculdade de Agronomia, Campus Do ValeAv. Bento Gonçalves, 7712 - Agronomia, Porto Alegre, RS Brazil; 3 Centro de Ciências Agrárias-CCA, Universidade Federal de Santa Catarina, Rod. Admar Gonzaga, 1346 Itacorub, Florianopolis, SC 88034-000 Brazil; 4 Animal and Poultry Production Department, Faculty of Agriculture, Minia University, El-Minya, 61519 Egypt; 5 Department Poultry and Fish Diseases, Faculty of Veterinary Medicine, Alexandria University, Edfina, El-Beheira 22758 Egypt; 6 Poultry Department, Agriculture Faculty, Zagazig University, Zagazig, 44519 Egypt

**Keywords:** Egg interior quality, Egg external quality, Minerals, Layers, Trace elements

## Abstract

With the advancement in the egg industry sector, egg quality has assumed great significance in certain countries. Enhancements in the nutritional value of eggs may have direct affirmative consequences for daily nutrient intake and therefore for human health. Thus, affirmative improvement in egg quality boosts consumer preferences for eggs. Also, the improvement in eggshell quality can avoid the disposal of broken eggs and consequently economic losses. Therefore, poultry nutrition and mineral supplements have a significant impact on egg quality. Minerals are crucial in poultry feed for a number of biological processes, including catalytic, physiologic, and structural processes. For instance, they contribute to the biological processes necessary for forming and developing eggshells. To produce high-quality eggs for sale, diets must therefore contain the right amount of minerals. This review aims to highlight the role of both organic and inorganic minerals in improving egg quality, in addition to reviewing the interactions of mineral supplements with intestinal microbiota and subsequent effects on the egg quality.

## Introduction

Egg is a good source of nutrients such as vitamins A, D, E, and K, B2, B6, B12, as well as minerals including calcium, phosphorus, selenium, zinc, iron, and magnesium [97]. Egg quality is a key criterion for egg producers all over the world and has significant economic ramifications. While poor egg quality can result in losses, improving quality can help increase the value of the finished product. The nutrition and digestive health of hens are directly related to this criterion. The rearing system of the hens significantly affects the concentration of nutrients including elements in the egg. Eggs from organic farming systems have richer nutrients like magnesium, calcium, and zinc compared with eggs laid from caged hens [97].

**Fig. 1:**
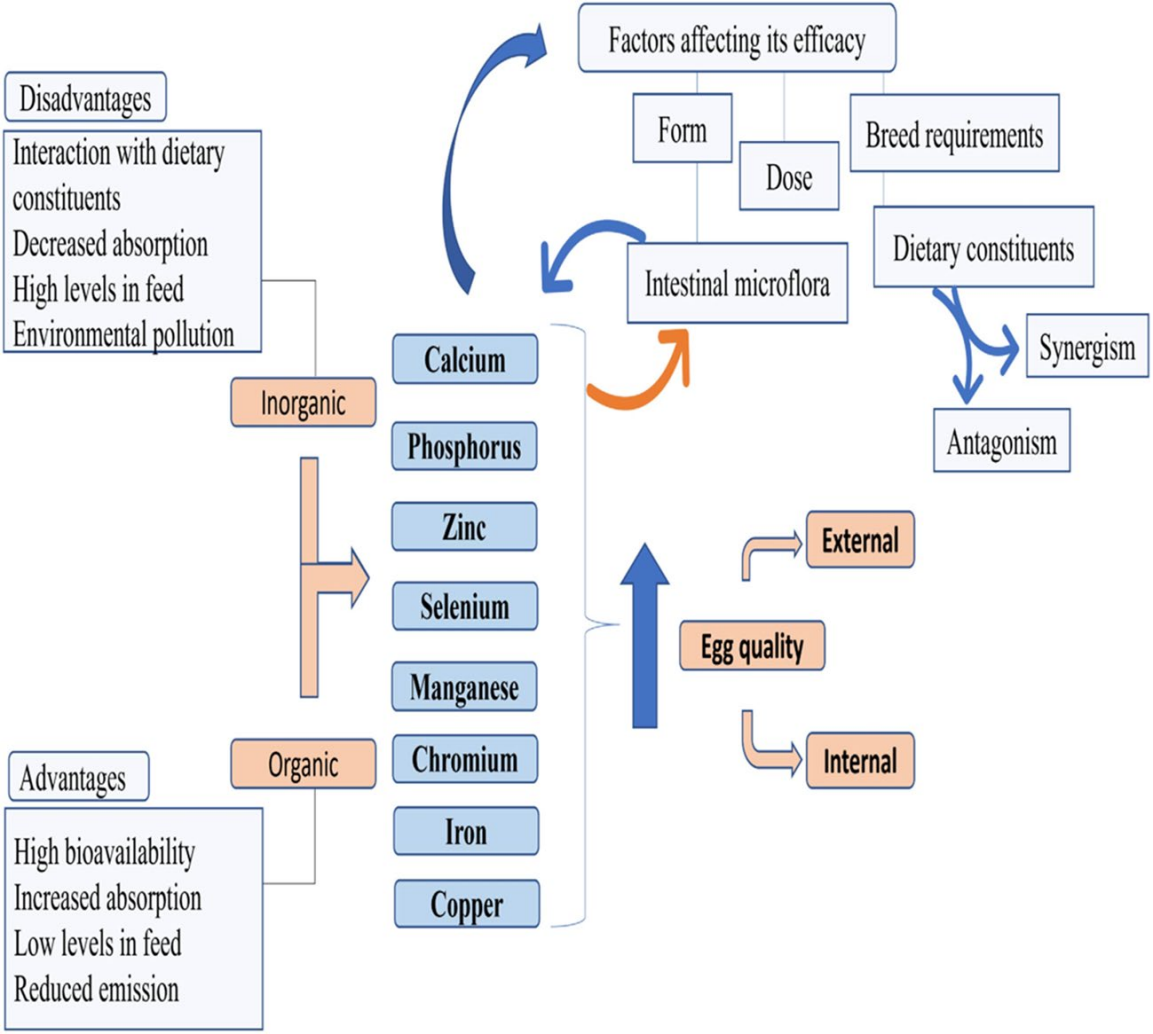
Effects of minerals in the laying hen nutrition on egg quality

Mineral supplementation in diets of layer chickens contributes to boosting egg quality criteria. Minerals are important for laying hen diets because they contribute to the biochemical processes that support normal development and growth of body and eggs [82]. Based on the National Research Council [69], it can be concluded that the total Zn, Mn, Se, I, Fe, Mg, and Cu requirement for laying hens is around 29–45, 17–25, 0.06, 0.32–0.48, 38–60, 370–600, and 4–5 mg/kg. Also, the requirement of sodium and potassium is around 0.13–0.19% in the laying hen diets. Maintaining the optimal concentration of minerals in diets is necessary to obtain high egg quality for sale [59, 61]. Incorporating minerals in layers’ feed is essential to produce superior external traits of egg quality and to diminish the problem of limiting levels of minerals in hen commercial diets which based on the ingredients of soybean meal and corn with low concentrations of minerals [32]. Mineral contents in the whole egg and eggshell are quite variable, according to the dietary element form and dose, as well as other aspects such as physiological reactions, management practices, geographic area, and supplemented feed additives [11, 37]. The consideration of the mineral source, organic or inorganic, that requires for supplementation must be taken into account. Dietary organic mineral supplementation significantly improved internal egg quality besides the external one [86]. Minerals can also enhance certain physiological reactions including the immune reaction [70] and play crucial roles in the virulence of pathogens and the antimicrobial resistance of hosts [110]. For instance, guanosine 5′-monophosphate-chelated calcium and iron inhibited the growth of *Salmonella gallinarum*, boosted percentage of egg production, and decreased the proportion of both cracked eggs and broken eggs [70]. Therefore, from another side, supplemental minerals can be used to improve the egg quality criteria. This review was aimed to summarize the benefits of mineral supplementation for laying hen diets, factors interfering them and to fill in gaps in the knowledge of the influences of dietary minerals on laying hens’ egg quality (Fig. 1).

## Minerals Sources in Diets of Laying Hens

Minerals are the main nutraceuticals required for physiological functions and optimum health. They are necessary as a part of the activator of enzymes and hormones for the eggshell formation and skeleton. The advanced knowing of the significance of minerals in reproduction and the changing of mineral levels in feed ingredients resulted in their supplementation in high quantities to the laying hens feed in the commercial sector with a considerable safety margin often surpassing the nutritional requirements [6, 7]. The inorganic sources are often incorporated into diets at higher proportions due to uncertainties associated with absorption (Araújo et al. 2008). However, this form results in a lack of nutritional balance and a potential for toxicological concerns [64], as well as a greater excretion of mineral than does the organic from [81, 102].

The bioavailability of minerals refers to the utilized portion by the organism, depending on their form (organic or inorganic). Inorganic minerals encompass oxides, sulfates, phosphates, and carbonates, while organic ones include proteinates, amino acids, polysaccharides, yeast, and complexes/chelates analogous to methionine. Feed-grade sources of trace minerals can differ greatly in purity. The variation in minerals’ bioavailability of these sources is also observed, wherein sulfates generally exhibit higher bioavailability than oxides [64]. Moreover, the element in some sources may need some enzymes to be available to the bird. For example, the bioavailability of P from plant sources is relatively limited, even though certain feed ingredients such as wheat and barley contain phytase which enhances the availability of P [14].

According to Suttle et al. (2010), bioavailability has been controlled by four steps: (1) the availability of such mineral to the absorptive enterocytes of mucosa that is affected by its form and its agonistic or antagonistic relations in the feed or in the gut; (2) the possibility of absorbed mineral transference through the mucosa layer (absorbability) which is depending upon the mucosa’s ability to uptake accessible minerals; (3) the ability of circulating minerals to avoid excretion through the kidneys or gut, and it measures the rate of mineral retention of the transferred ones (retainability); (4) the capacity of the minerals that assimilated into functional forms of retained ones which is influenced by the mineral absorbed forms and the site of its retention.

Organically chelated minerals showed superior bioavailability than those of inorganic metal salts [66, 101],therefore, using chelated or organic complexes of minerals in layers’ diets has been reported. The bioavailability of trace minerals determines their efficacy. Therefore, it appears that the additives of minerals in organic are more effective in supporting criteria of egg quality and egg production in laying hens [24, 32, 92, 106]. Furthermore, the complete substitution of inorganic mineral sources with organic ones in laying hens’ diets allowed the decrease in excreted minerals and did not impact egg production or the eggshell quality [20, 21]. Trace metal-amino acid complexes have the potential to mimic the mechanism by which trace elements are absorbed, making them available to animals more than inorganic forms [95]. However, such complexes must be robust enough to withstand natural dietary antagonists while still providing the complexed element to the tissues in a form that can be utilized [95]. The role of some minerals in enhancing egg quality will be shown in this review along with the difference between the effects of organic forms of minerals versus inorganic forms in laying diets (Table 1).

**Table 1:** Effects of the mineral supplement source and its doses on egg quality in laying hens

Breed	Age (weeks)	Supplementation period (weeks)	Mineral content in basal diet (BD)	Mineral supplement	Main results	Reference
Hy Line W36 white hens	69	16	0.250 kg/ton of: IM (Zn, Mn, and Se at 50 g, 65 g, and 100 mg/kg product, respectively)	0.250 kg/ton and 0.50 kg/ton of OM: (Zn, Mn, and Se at 30 g, 30 g, and 300 mg/kg product, respectively)	√ Cracked and thin shell eggs%, eggshell%, total egg solids and fresh and dried yolk yields > BD ↕ Eggshell thickness, specific gravity, Haugh unit (HU), albumin %, and solids	Fernandes et al. [30]
Hy-line brown semi-heavy layers	83	16	-	OM replacing IM by 110, 100, 90, 80, 70% IM: Zn, Fe, Mn, Cu, I, and Se at 54, 54, 72, 10, 0.61, 0.3 g/kg, respectively OM: Zn, Fe, Mn, Cu, I, and Se at 30, 30, 40, 6, 0.61, and 0.3 g/kg, respectively	√ Egg specific gravity and eggshell % by replacing 80% of IM by OM ↕ Egg quality, yolk%, and albumen% by OM	Saldanha et al. [85]
Hy-line W-36 hens	(44 to 80 weeks of age)	56	-	IM and OM of Zn–Cu–Mn M0: 0–0-0 ppm IM1: 20–5-20 ppm OM1: 20–5-20 ppm IM2: 40–10-40 ppm OM2: 40–10-40 ppm IM3: 80–10-80 ppm	↓ Shell thickness by M0 or IM1 > IM3 # OM1 and IM3 √ OM1 > IM1 √ Shell breaking strength, shell thickness by OM > IM	Manangi et al. [62]
Barred rock hens	50	16	Mn, Zn, Cu, and Cr at 12.3, 31.9, 5.3, and 0.88 mg/kg, respectively	OM or IM: 0.05% (Mn, Zn, Cu, and Cr at (40, 30, 2.5, and 0.07 mg/kg, respectively) 0.1% (Mn, Zn, Cu, and Cr at 80, 60, 5, and 0.15 mg/kg, respectively	√ Concentrations of egg Mn, Zn, Cu, and Cr; and eggshell Zn and Cr by OM > IM ↓ Mn, Zn, Cu, Cr, and Ca excretion by OM ↕ Cr and Ca excretion by addition level √ Egg Mn, Zn, Cu, and Cr; and eggshell Mn, Zn, and Cu concentrations by high dose > low dose	Yenice et al. [113]
Bovans white hens	60	12	Fe, Zn, Cu, Mn, Se and I at 120, 23, 12, 53, 0.15 and 0.15 ppm, respectively	400 ppm of IM (, Zn, Mn, Fe, Cu, I, and Se at 7, 70, 45, 10, 1 and 0.25 mg/kg, respectively) 700 and 1000 ppm of OM (Zn, Mn, Fe, Cu, I, and Se at 43.74, 56.37, 43.74, 8.61, 1, and 0.34 mg/kg, respectively)	√ Yolk color; shell breaking strength; and storage time by 700 ppm OM > IM # OM and IM on dirty or broken eggs, HU, and shell thickness	Ramos‐Vidales et al. [80]
HY-line white hen	50	8	Fe, Cu, Mn, and Zn at 36, 12, 90, and 90 mg/kg, respectively (commercial level)	IM: commercial level and at 1/3 commercial level OM: at 1/3 commercial level	↓ Albumen height, eggshell strength, and yolk Fe concentration by 1/3 dose of IM ↓ Fecal mineral excretion without negative effects on egg quality by OM	Qiu et al. [77]
HY-line white hen	50	8	Fe, Cu, Mn, and Zn at 36, 12, 90, and 90 mg/kg, respectively (commercial level)	IM1: at commercial level IM2: at 1/3 commercial level OM: at 1/3 commercial level	↓ Eggshell strength, eggshell palisade layer, palisade layer ratio, and carbonic anhydrase activity by IM2 > IM1 √ Egg loss and mammillary layer ratio IM2 > IM1 # OM and IM1 for all the indices ↓ Mammillary knobs size by OM > IM1 and IM2	Qiu et al. [78]
Hy-line brown hens	68	5	Fe, Cu. Zn, Mn, and Mg at 92.3 mg, 12.9 mg, 24.2 mg, 22.5 mg, 2115 g/kg, respectively	g/kg of IM 1.8 g/kg of OM (Fe, Cu, Zn, Mn, and Mg at 191 g/kg, 174 g/kg, 180 g/kg, 168 g/kg, and 90 g/kg, respectively)	√ Eggshell strength by OM > IM and BD √ Broken and shell-less eggs% by IM and OM > BD ↕ HU, eggshell thickness and color, egg weight, and egg yolk color between treatments	Kim et al. [46]
Jinghong-1 laying hens	57	8	Cu, Mn, Zn, and Fe at 6, 29, 49, and 308 mg/kg of diet, respectively	IM (100%): 10, 80, 80, and 60 mg·kg^−1^ of Cu, Mn, Zn, and Fe, respectively OM1: by fifth dose of IM OM2: by third dose of IM OM3: by half dose of IM	√ or # Cu, Mn, Zn, and Fe deposition in egg yolks by OM1, OM2, or OM3 > IM √ Eggshell breaking strength and the antioxidant status of the eggshell gland by OM3	Dong et al. [24]
Jing Hong hens	57	8	Cu, Zn, Mn, and Fe at 6.76, 29.11, 49.77, and 308.20 mg/kg, respectively	IM: 100% (Cu, Zn, Mn, and Fe at 10, 80, 80, and 60 mg/kg) OM: replacing IM by 20%, 30%, or 50%	√ Eggshell color by OM 50% > IM 100% # Eggshell breaking strength and ratios by OM 50% and IM 100% √ Mineral deposition in the eggshells by IM and OM > BD and by increasing doses of the OM	Zhang et al. [117]
White Plymouth rock hens	36	12	10 mg Cu, 60 mg Fe, 70 mg Mn, 75 mg Zn, and 0.3 mg Se/kg of diet	500 g/ton 800 g/ton of OM: (5 mg Cu, 35 mg Fe, 40 mg Mn, 55 mg Zn, and 0.16 mg Se/kg of product)	↕ Egg, yolk, albumen, and eggshell weight, specific gravity, HU, yolk and albumen pH, eggshell thickness, and shell strength ↓ Yolk index by 800 g OM > BD	Londero et al. [56]

*IM*, inorganic minerals; *OM*, organic minerals; √, has an improved effect; ↕, has no effect; ↓, has decreased effect; #, has comparable response; *BD*, basal diet

## Effects of Minerals on Egg Quality

### Zinc (Zn)

Zinc performs multiple functions in the formation of the skeletal system, the regulation of metabolic processes, the maintenance of antioxidant systems, and the enhancement of the immune response in poultry, as well as egg formation [6, 7]. Zinc is a constituent of several metalloenzymes, for example, carbonic anhydrase, which has crucial role in eggshell formation [116], where the crystal and texture morphologies of the eggshell are affected by carbonic anhydrase’s catalysis of carbon dioxide into bicarbonate ions [51]. Zinc is a vital trace mineral necessary for forming eggshells and can contribute to the process of calcium deposition as well as impact the structure and physical properties of the shell [51, 65]. Zn serves as a significant cofactor in carbonic anhydrase enzyme which is responsible for facilitating the hydration of circulating CO2 into HCO3 − and providing the precursor for eggshell carbonates [122]. Zinc is important during albumen deposition in the magnum, eggshell membrane formation in the isthmus, and shell formation in the uterus [12]. Augmented Zn supplementation may decline egg loss from breaks and cracks. Several research has demonstrated superior eggshell thickness and strength and less egg breakage in birds administrated with Zn in organic form or in a combination of inorganic and organic forms [40, 71, 92]. The addition of zinc methionine chelate to layer diets is recommended because it results in improved bone mechanical properties, without compromising the quality of eggshell at the end of the egg-laying cycle, indicating its positive impact in the overall maintenance of bone mineral reserves through the end of the egg-laying cycle [70].

Mixing ZnSO_4_ and Zn-amino acid (ZnAA) complex in broiler breeder feeds diminished the percentage of cracked eggs and enhanced the eggshell quality in comparable to the basal diet that contained ZnSO4 [40]. Swiatkiewicz and Korelski [96] illustrated that 50 and 100% substitution of Zn oxide with ZnAA complex increased the eggshell breaking strength in the aged hens (62:70 weeks of age) that received the basal diet enriched with 30 mg Zn/kg. Moreover, Manangi et al. [62] exhibited that using Zn–Cu–Mn chelated with hydroxy analog of methionine (20–5-20 and 40–10-40 ppm) as an organic Zn supplement augmented both eggshell strength and eggshell thickness in layers (44 to 80 weeks of age) comparing to those consumed the basal diet only or the basal diet enriched with sulfate salt of Zn–Cu–Mn (20–5-20, 40–10-40, and 80–10-80 ppm). Thus, Zn might ameliorate the adverse influence of age on the quality of eggshell.

Li et al. [50] stated that adding Zn-methionine (Zn-Met) in the basal diet at level of 100 ppm augmented egg’s Haugh unit (HU) and albumen height in comparable to the control that fed the basal diet supplemented with 80 ppm Zn as sulfate salt. Abd El-Hack et al. [1] illustrated that supplementing diets with 50, 75, or 100 ppm Zn-Met had considerable affirmative influence on the HU compared to the control with no Zn-Met supplements. These enhancements may be owing to the significance of Zn function in the egg formation. Besides, Amen and Al-Daraji [9] pointed out that the epithelium quality is affected by the deficiency of Zn owing to its role in the synthesis of protein.

As previously mentioned, zinc has an indirect impact on the secretion of the epithelium layer by altering its structure or directly influencing the secretion of the eggshell membranes. Also, it plays a vital role in the magnum and isthmus during egg albumin and eggshell membrane formation, respectively. Tabatabaie et al. [98] have demonstrated that HU and egg albumen percentage were elevated by dietary administration of 25 or 50 ppm organic Zn, in comparison to the control with no Zn administration. Nevertheless, Idowu et al. [41] declared no statistical changes in criteria of egg quality, except the values of HU, when layers consumed diets enriched with Zn-proteinate, Zn-carbonate, Zn-oxide, and Zn-sulfate at a level of 140 ppm. Also, zinc could maintain the quality of the stored eggs due to its ability to activate enzymatic antioxidant system in the egg. Organic zinc, at a concentration of 60 to 80 ppm, exerted a more pronounced influence in this regard compared to 80 ppm inorganic zinc [50]. According to Zhao et al. [144], zinc has an anti-oxidation function that makes birds more resistant to certain oxidative stresses. Furthermore, Zn can be used as a dietary supplement to control the negative effects brought on by various agents such as aflatoxicosis in addition to serving as a nutrient for birds [67]. Generally, eggshell traits of layers could be improved by the replacement of inorganic Zn with organic one, especially in older birds, but the mechanism was still unclear.

### Selenium (Se)

Selenium revealed strong biological and nutritional influences in inducing physiology and production of poultry [6, 7] that were primarily mediated by the activity of selenoproteins [55, 93]. The dietary requirement of selenium for laying hens is comparatively low, at approximately 0.3 mg/kg [79]. Excess Se intake is toxic [101], and its inorganic form has limited biological availability, consequently its inclusion by high doses limits its utilization in poultry nutrition [120], as well as its emission to the environment is higher than the organic form [117]. Organisms take inorganic selenium and turn it into organic form [94]. Selenium-enriched yeast culture, bacterial Se, SeMet, OH-SeMet, Se-cysteine, and semethyl-Se cysteine are examples of selenium organic sources. Organic form of selenium can be transferred to eggs [18] with a higher rate than inorganic Se through its addition to diets of egg-laying hens. In addition to the enhanced efficacy in transferring to eggs, a better internal egg traits (e.g., HU) was documented as a result of the stimulation of selenoprotein, methionine sulfoxide reductase B enzyme, which is demanded for preventing protein oxidation and maintaining the albumen water-holding capacity [42, 102].

The dietary supplementation level of Se yeast (0.21, 0.36, and 0.43 ppm) increased Se levels in the whole egg, albumen, and yolk of laying ducks when compared to control feed that contained Se at level of 0.15 ppm [118]. A positive correlation (linear and quadratic) has been observed between the concentrations of Se in the egg and the level of Se-enriched yeast in the diet (0.3, 1.5, and 3 ppm) of 30-week-old laying hens [58]. However, after a 12-week feeding period, no considerable differences were noted in the fresh egg quality criteria (external and internal) between hens that received Se-enriched yeast diet and those fed a basal diet with no Se supplement.

Selenium is a vital component in many antioxidant enzymatic systems; consequently, the addition of Se might boost the activity of GSH-Px [107] and total antioxidant status of eggs when compared to the control with no Se addition [79].

Selenium can be used to prolong the storage period of the eggs due to its antioxidant effects. Saldanha et al. [85] stated that higher egg Se content allows maintaining the internal quality of egg during storage. Enriching layers diets with selenium markedly augmented its concentration in the egg, fatty acid composition, oxidative stability, and maintains quality of stored eggs such as yolk index [25, 35]. The greatest benefit regarding egg oxidative stability was seen by organic selenium addition in diets high in oxidized fat sources (Laika and Jahanian 2015).

Muhammad et al. (2021) recorded differences among organic (yeast and bacterial) and inorganic Se in gene expression of GPX1, GPX4, DIO1, DIO2, and SELW1, and they attributed it to the superior bioavailability of organic forms, which stimulates more selenoprotein gene expression [105]. It is notable that organic Se benefit is beyond just improved absorption. For instance, chelated Se to amino acids has much retention and incorporation into animal tissues [17] which might act as amino acid analogs for creating non-specific proteins. Additionally, the absorption and transportation of selenium in cleated form may be accomplished entirely to the target tissues, ultimately leading to higher bioavailability for metabolism when compared to inorganic selenium [17]. However, organic Se from different sources possesses different bioavailabilities in the body. So, the efficacy of organic Se source and level on layers’ performance and egg quality should be investigated.

Given that laying hens’ table eggs are used as food with high levels of selenium or as raw materials for food that are enriched with selenium [57], it follows that adding organic selenium in layer diets has remarkable practical importance for consumers.

In comparison to its inorganic counterpart, organic selenium is less toxic, has higher bioavailability and rates of retention and tissue accumulation, and possesses antioxidant properties. However, genome instability may result from excessive Se intake due to oxidative damage. Therefore, further examining the clinical and safety parameters of organic sources of Se and levels in healthy laying hens should be investigated.

### Manganese (Mn)

Manganese plays a significant role in bone development of layers and is necessary for forming of eggshell and can positively affect the quality of eggshell [72, 104]. Manganese boosted eggshell strength by augmenting the process of biosynthesizing glycosaminoglycan (GAG) [121], which regulating mineral deposition eggshell and consequently determining the quality [22]. Supplementing the diet with Mn has the potential to enhance the quality of eggshells through the augmentation of GAG and uronic acids in the eggshell membrane [109]. The presence of Mn can influence the mechanical properties of the eggshell by modulating the formation of calcite crystals and the structure of the shell [59, 96]. Mn serves as a stimulator for enzymes involved in synthesizing mucopolysaccharides and glycoproteins, both of which play a crucial role in the creation of the organic matrix that forms the shell [86]. In the review of Olgun [72], the laying hens’ performance seems to be unaffected by dietary supplementation of inorganic Mn at dose of 200 ppm, but at lower doses, eggshell quality is improved. It appears that laying hens need about 90 ppm of Mn in their feed, and Mn-sulfate is more readily available than other forms of inorganic Mn, but lower than its organic forms. Mn is capable of activating enzymes that participate in the creation of glycoproteins and glycosaminoglycans, both of which participate in forming the shell organic matrix [92]. Sazzad et al. [87] found that eggshell thickness was augmented with the increase of dietary Mn addition up to 105 ppm (80 ppm MnO + 25 ppm basal diet). Fassani et al. [29] pointed out that elevating dose of dietary Mn supplementation (40 to 200 ppm) in the second production phase linearly augmented shell thickness and egg loss rate of laying hens. Mn deficiency causes a reduction in egg yield, enhanced the formation of eggs with a thin shell with translucent regions, and exhibited abnormal ultrastructure of the eggshell [33, 59].

Xiao et al. [108] noted that adding 100 ppm Mn in laying hen diets boosted eggshell characteristics (break strength, fracture toughness, and thickness), increasing uronic acid and glycosaminoglycan formation in the uterus, and consequently boosting the shell ultrastructure compared to basal diet with no Mn supplement. The use of Mn-Bioplex (organic form), at different levels in diet (15, 30, 45, 60, and 75 ppm) for 12 weeks in laying hens, increased the egg weight and decreased the of broken eggs %, when compared to the inorganic form [114]. Li et al. [49] studied the impact of dietary MnSO_4_ (60 ppm) as a control group and dietary Mn-methionine (Mn-Met) (20, 40, 60, and 80 ppm) on ​​egg quality in laying hens and found that dietary Mn-Met treatments improved the egg internal traits (yolk color, albumen height, and HU), and the ultrastructure of the shell. Eggshell Mn levels were significantly increased by increasing Mn-Met addition, indicating that Mn content was distributed mainly in the eggshell. These studies demonstrate that the mechanical properties of eggshells were improved by dietary Mn.

Previous research has indicated that dietary supplementation of Mn with either organic or inorganic Mn can enhance the production and eggshell quality in aged laying hens and deficiency of Mn could potentially decline the shell matrix content of hexosamine and hexuronic acid [116]. This deficient in Mn causes a notable decrease in the levels of GAG and uronic acids within the shell membranes, but it does not in the calcified shell [109]. In addition, manganese deficiency in the diet led to diminish the expression of GlcAT-I mRNA and inhibit the synthesis of GAG in the uterus [109]. In the future, it might be necessary to reevaluate the requirements Mn for layers in order to achieve optimal eggshell characteristic.

### Copper (Cu)

Copper plays a significant role in forming shell membranes, which influence eggshell texture, shape, and structure [34]. Copper has been detected at high levels in eggshell and its membranes. The deficiency of dietary copper has the potential to impact the shell membrane structure, texture, and shape, and the pigments of the eggshell [75]. Copper possesses the capability to impact the quality of the eggshell by stimulating the enzymes that involved in the processes of forming eggshell and its membrane, in addition to their ability to interact with calcite crystals during the eggshell formation process [33]. Copper is a vital element of the lysyl oxidase enzyme, which being important in forming collagen of eggshell membrane [48]. Copper ion is an active cofactor in the center of superoxide dismutase, an enzyme that inhibits free radical reactions. Furthermore, copper reduced yolk cholesterol content [54] and increased shell strength [76]. Dobrzañski et al. [23] confirmed that using organic Cu significantly increased the Cu content in eggs and eggshells, which indicates the higher availability of organic Cu compared to CuSO_4_. Lim and Paik [53] concluded that dietary supplementation of 100 ppm of methionine-Cu chelate can enhance eggshell quality compared to the control diet with 20 ppm of Cu. Olgun et al. [73] obtained eggs with lower percentage of broken eggs and heavier and thicker shells in birds supplemented with Cu (75 to 300 mg/kg feed) than no Cu supplementation. Pekel and Alp [76] stated that adding dietary organic copper exhibited no statistical changes in egg quality characteristics or yolk cholesterol. However, shell strength was decreased in eggs from layers supplemented with micronutrients including inorganic and organic Cu forms. Cu-lysine chelate supplementation in the drinking water (30 mg/L) of laying hens improved egg weight and albumen weight and height, with no changes in the shell strength parameters compared to control birds with no additives [20].

The deficiency of copper in hen diets leads to the occurrence of abnormalities in eggshell [75]. The eggshell of hens suffering from copper deficiency showcases an unconventional arrangement of the fibers of shell membrane, due to changes in the cross-links derived from lysine. This ultimately causes abnormalities in egg shape and physical properties [26]. Furthermore, the lack of copper as micronutrient, which being a component of numerous enzymes and their activators, can reduce egg yield and heighten the frequency of eggs with abnormalities in size and shape. For the best egg yield and quality, it is therefore recommended to determine the minimal effective dose, Cu form and source, and the timing of administration.

### Iron (Fe)

Iron is an important cofactor of many enzymes and acts in the oxygen transporting and storing. It is involved in protein and energy metabolism, and improving antioxidant and immunity status [6, 7]. Iron participates in several important reactions such as oxygen transporting and storing, as well as energy supply and protein metabolism that controls egg production [60, 110]. Seo et al. [88] stated that providing diets with 100 ppm iron enhanced formation and breakdown of erythrocyte, and boosted egg color in brown-type hens through its role in the protoporphyrin production (the main shell brown pigment). Compared to the Fe depletion pretreatment group, Fe increases egg production and blood hemoglobin without changing the eggshell color and egg composition [105]. Xie et al. [111] revealed that dietary treatments with Fe-glycine (Gly) (20, 40, 60, and 80 ppm) improved internal egg quality (albumen height and HU) in comparable to control that received 60 ppm Fe as FeSO4. Nevertheless, dietary supplementation of Fe-Gly showed little effect on eggshell ultrastructure. Shell, yolk, and albumen contents of Fe heightened by dietary level Fe-Gly, where dietary Fe-Gly (60 or 80 ppm) showed greater content of iron in albumen and yolk than control. Bertechini et al. [19] noted higher concentrations of iron in the egg when the basal diet enriched with 80 ppm Fe as FeSO4. Also, Paik [74] detected that utilization of chelated Fe augmented the iron content of the yolk by up to 20%.

Egg enrichment with minerals could be achieved by dietary trace element supplementation. In addition, iron is one of the more significant trace elements for poultry, playing a vital role in egg production and quality. Therefore, in order to enhance these parameters, a suitable concentration of Fe must be provided.

### Chromium (Cr)

Chromium has important functions in the metabolism and antioxidant status of birds [6, 7]. The legality of adding Cr to an animal’s diet differs depending on the country, the source of the Cr, and the kind of animal [90]. In 2016, the FDA approved the use of 200 ppb of chromium in the whole feed of broiler chickens [31]. The European Food Safety Authority recommended using 0.4–1.6 mg/kg of chromium methionine as a feed additive in full feeding mixes for all species (EFSA 2009).

Chromium has been detected as component in egg albumin and in protein cross-linking, which is required for creating albumen proteins and aides in transporting the ion to the egg albumin throughout the plumping process in the shell gland [44]. Moreover, it is hypothesized that the presence of chromium is essential for maintaining the physical properties of albumin [44]. Dietary chromium supplementation (400 ppb to the basal diet contained 1285 ppb) linearly increased the HU and shell thickness of layer chickens kept at low temperatures compared to the control diet [84]. Lien et al. [52] displayed no changes in the shell thickness in response to supplemental Cr picolinate at dose of 200, 400, or 800 ppb under thermally neutral conditions. Similarly, in 24- to 33-week-age brown-type layers, supplemental Cr picolinate in diets at 400 or 600 ppb had negligible effect on shell thickness and strength, yolk color, and HU compared to non-supplemented Cr group. Torki et al. [100] observed greater weight and eggshell thickness of heat-stressed hens that received 400 ppb of Cr picolinate compared to the Cr-non-supplemented control birds. Subsequently, Torki et al. [99] pointed out that adding Cr to diets of laying hens exposed to heat stress increased the HU and shell weight of eggs produced from laying hens after exposure to heat stress. Nevertheless, supplemental Cr exhibited insignificant impact on internal egg quality. These studies demonstrate that chromium supplementation is important to improve the shell quality of eggs produced at non-neutral temperatures.

## Effect of Nano-minerals on Egg Quality

Lately, the utilization of nano-minerals has garnered considerable interest owing to their elevated bioavailability; hence, their integration in poultry diets is capable of augmenting performance and health [2, 27, 38, 118] (Table 2). Incorporating nano-minerals in diets positively affects egg quality of laying hens [47]. The incorporation of selenium nanoparticles into the layer feeds exhibited a remarkable impact on the egg laying productivity, egg quality, and the activity of oxidative enzymes [68]. Sirirat et al. [89] stated that using chromium picolinate nanoparticles has the potential to enhance the quality of eggs. The latest authors revealed that layer-fed diets containing 500 and 3000 ppb nanometric chromium picolinate produced eggs with low yolk weight percentage. Laying hens fed on diets supplemented with zinc oxide nanoparticles achieved the highest significant improvement in egg shell quality, egg index, yolk and white quality, and HU compared to the control group [4]. The enhancement observed can potentially be attributed to the exceptional characteristics of nano-minerals, e.g., their remarkable biological availability, higher surface activity, improved mobility, increased solubility, and enhanced cellular uptake [63, 115].

**Table 2:** Examples of interaction of dietary constituents with minerals and subsequent effects on egg quality

	Dietary item and exposure form	Interaction effect	Reference
Negative effect	Prolonged exposure to Cr	Decreased the serum levels of Ca, Fe, Mg, Zn, and Cu	Zhu et al. [122]
	Zn deficiency along with supplementation of organic Cu, Fe, and Mn alone or in combination	Did not significantly improve bird performance and were primarily excreted	Bao et al. [15]
	More Zn and Fe intake	Decrease the availability of Cu	Gheisari et al. [33]
	Inositol phosphate 6 (InsP6): organic P source	Is anti-nutritional factor forming complexes with minerals and inhibits the absorption of cations	Woyengo and Nyachoti [108]
	High calcium content	Inhibit phytate-degrading enzymes, reducing the hen’s access to phosphorus	Hofmann et al. [39]
	Zeolite inclusion in the diet (anti-mycotoxin agent)	Significantly increased serum concentrations of Zn and Al and decreased P, Mg, and Cu, without effect on the Ca concentration	Utlu et al. [103]
Positive effect	Phenolic compounds in plant extracts and essential oils	Are powerful chelating agents for iron and copper, enhancing their absorption	Andjelkovic et al. [10]; Garcia et al. [32]
	Exogenous phytase	Improves the zinc digestion	EFSA [28]

## Interaction of Dietary Constituents with Minerals and Subsequent Effects on Egg Quality

The inorganic trace mineral absorption is limited by the interactions with other feed nutrients including the other trace minerals [111]. The mineral form and quantity determine how much mineral is deposited in the egg contents [13]. For example, chickens exposed for a long time to Cr showed drops in serum levels of Ca, Fe, Mg, Zn, and Cu [122]. Zinc is considered the first limiting trace mineral among others, i.e., Cu, Fe, Mn, and Zn, so supplementing Zn-deficient diets with organic form of these minerals alone or in combination led to nonsignificant change in bird performance and were primarily excreted [15]. Zn-methionine exhibits superior bioavailability compared to inorganic one owing to its stability and withstands interference against disruption caused by other ligands in the gastrointestinal system [51]. The availability of Cu could be decreased by consuming more Zn and Fe, and a lack of Cu would cause abnormalities in shell formation [33].

The most significant source of organic P, inositol phosphate 6 (InsP6), is regarded as an anti-nutritional factor because it forms complexes with minerals and inhibits the absorption of cations in poultry [108]. The high-calcium-content diets of layers, which is necessary for forming eggshells, may inhibit phytate-degrading enzymes, reducing the hen’s access to phosphorus [39] and additionally resulting in the inappropriateness of phytase superdosing in mixed diets of layers (Skřivan et al. 2018).

On the other hand, the synergy between minerals and other dietary supplements, e.g., phenolic compounds and essential oils that present in plant extracts, can maximize the effects of minerals. The chelation among these compounds augments the absorbed minerals and their utilization; this shows that the two together can be more effective than either used alone [91].

Dietary incorporation of inorganic mineral with 100 ppm of rosemary oil enhanced yolk color and the relative weight of egg albumen of laying hens [32]. Additionally, the advantages of using essential oils on digestibility [8] led to more absorption of nutrients, including minerals, from the feed, and enhancing the quality of eggs. A further strategy to lessen the negative effects of P, Cu, and Zn on the environment is to increase mineral digestibility using exogenous enzymes. For instance, exogenous phytase added to the feed improves the piglets’ ability to digest zinc [28]. Additionally, this would require less mineral supplementation ([28]; EFSA 2016). Therefore, research into the adverse effects and interferences of trace minerals, particularly in mixtures, on digestion, absorption, and utilization, should be done in order to spot nutritional solutions to maximize egg yield and quality while reducing the environmental pollution.

## The Interaction of Intestinal Microbiota with Minerals and Subsequent Effects on Egg Quality

A complex community of numerous microorganisms makes up the gastrointestinal tract (GIT) microbiota of laying hens. This microbial community creates the intestinal microbial barrier, which is crucial for nutrient digestion and absorption, gut health, and the immune system [5, 45]. There are differences in microbial composition that can be seen related to feed and its nutrients (Leeming et al. 2021). A two-way relationship exists between trace minerals and the digestive tract microorganisms. Through their ability to directly affect the absorption of minerals within the digestive tract and the production of important enzymes like phytase, which assist in releasing minerals from the feed, microorganisms of gastrointestinal tract exert a profound influence on the metabolic processes of trace minerals [36]. Additionally, an acidic medium and strong gastrointestinal functions are necessary dissolving calcium and phosphorus to make them more absorbable and utilizable [112]. On the other way, the gut microbial content and role might be changed by trace minerals, and they could also compartmentalize metabolic inflammation [16].

## Mineral Supplementation May Change Gut Microbiota Composition, Resulting in Improved Egg Quality

According to Dong et al. [24], the harmful cecal microbiota (Barnesiellaceae and Clostridia) significantly decreased in layers that consumed inorganic (full dose) or organic (half dose) Cu, Mn, Zn, and Fe when compared to control. Furthermore, they demonstrated an association between the gastrointestinal microbiota and the genes’ expression associated with the uterus (such as vocalyxin-32 (OCX-32), osteopontin (OPN), and ALAS). As a result, distinct microbiota was negatively correlated with various eggshell characteristics, including absolute and relative weight, quality, and breaking strength. Hence, the integrity gastrointestinal tract and higher absorbability of trace minerals may be related to the decrease in the load of dangerous Barnesiellaceae and Clostridiales bacteria. But the thickness and color of the eggshell, however, were not connected to any cecal bacteria [24]. According to Abdelqader et al. [3], supplementing diets of laying hens with *Bacillus subtilis* exhibited higher relative weight of the eggshell and higher calcium retention, along with a decline in the load of intestinal Clostridium. Adding organic yeast Se to laying hen feed (0.3 mg/kg) increased the diversity of intestine microbes in pre- or post-challenge with *S. Enteritidis*, maintained gastrointestinal well-being by raising the load of anti-inflammatory microbes (Barnesiella and Bacteroidales), and enhanced the egg quality [43]. According to the authors, selenium supplementation exhibited considerable influence on how the content of the gut’s microbes responded to antioxidants or immune stimulation. In order to enhance the quality of eggshells and hen health, intestinal bacterial regulation can therefore be crucial. However, Roth et al. [83] demonstrated that the distribution of the microbiota and predicted functions in two breeds of high production laying hen were unaffected by a 20% reduction in dietary Ca and P.

## Conclusion

Using organic minerals in the nutrition of laying hens has a wide range of advantages that apply to different product categories. To achieve the beneficial potential of supplementing feeds of laying hens with mineral, it is required to pay attention to the different supplementation doses, the different sources, their bioavailability, and transport as well as understanding the mechanisms to obtain much greater bioavailability. The possibility of using reduced mineral doses from organic sources could improve egg quality and reduce Cu, Zn, and P emissions into the environment. However, the output of mineral supplementation in laying hen diets is affected by the strain, breed, age, or duration of supplementation, and interactions between trace elements and feed additives. Only the optimal mineral supplementation would support high productive and reproductive performance and egg quality (internal and external traits). To understand the mechanisms of augmentation or antagonistic effects and achieve the greatest beneficial effects for laying hens, it is possible to focus on the interaction of probiotics or the microbiome in general with mineral supplementation. In addition, the efficacy within the different types of organic minerals (complexes/chelates) and their mechanisms in laying hens should be highlighted in further studies. Some trace elements have antioxidants or immune-stimulating activities, such as zinc, selenium, copper, and iron have direct and indirect positive effects on laying birds. They are not only used as nutrients to support egg performance but they also play a significant protective role against the negative effects of stressful situations, such as illnesses that affect egg production and egg quality, or they can strengthen various body systems against these agents. Data concerning the organic minerals’ impact on egg quality traits are quite variable and molecular mechanisms of organic mineral actions need further research. In addition, substantially increased cost, in comparison to inorganic minerals, is an important factor to be considered in commercial poultry production. Therefore, it is crucial to assess the mineral requirements for laying hens to be advantageous on a variety of aspects of the bird’s body under healthy or disease conditions by choosing the ideal dose and form without having negative effects on the bird, its products, and subsequently the consumer, or the environment.

## Data Availability

The datasets used and/or analyzed during the current study are available from the corresponding author on reasonable request.
